# CLytA-DAAO Chimeric Enzyme Bound to Magnetic Nanoparticles. A New Therapeutical Approach for Cancer Patients?

**DOI:** 10.3390/ijms22031477

**Published:** 2021-02-02

**Authors:** María Fuentes-Baile, Elizabeth Pérez-Valenciano, Pilar García-Morales, Camino de Juan Romero, Daniel Bello-Gil, Víctor M. Barberá, Álvaro Rodríguez-Lescure, Jesús M. Sanz, Cristina Alenda, Miguel Saceda

**Affiliations:** 1 Unidad de Investigación, Fundación para el Fomento de la Investigación Sanitaria y Biomédica de la Comunidad Valenciana (FISABIO), Hospital General Universitario de Elche, Camí de l’Almazara 11, Elche, 03203 Alicante, Spain; fuentes_marbai@gva.es (M.F.-B.); m.juan@umh.es (C.d.J.R.); barbera_vicjua@gva.es (V.M.B.); 2 Departamento de Bioquímica y Biología Molecular, Instituto de Investigación, Desarrollo e Innovación en Biotecnología Sanitaria de Elche (IDiBE), Universidad Miguel Hernández, Avda, Universidad s/n, Ed. Torregaitán, Elche, 03202 Alicante, Spain; elizabeth.perez@goumh.umh.es (E.P.-V.); pgarcia@umh.es (P.G.-M.); daniel.bello@remabtx.com (D.B.-G.); 3 Unidad de Genética Molecular, Hospital General Universitario de Elche, Camí de l’Almazara 11, Elche, 03203 Alicante, Spain; 4 Servicio de Oncología, Hospital General Universitario de Elche, Elche, 03203 Alicante, Spain; arodriguez@umh.es; 5 Centro de Investigaciones Biológicas Margarita Salas (Consejo Superior de Investigaciones Científicas) and Centro de Investigación Biomédica en Red de Enfermedades Respiratorias (CIBERES), C/Ramiro de Maeztu, 9, 28040 Madrid, Spain; jmsanz@cib.csic.es; 6 Unidad de Investigación, Instituto de Investigación Sanitaria y Biomédica de Alicante (ISABIAL), Hospital General Universitario de Alicante, C/Maestro Alonso, 10, 03010 Alicante, Spain; alenda_cri@gva.es

**Keywords:** enzymatic therapy, reactive oxygen species, magnetic nanoparticle, gold nanoparticle, alginate capsules, hydrogen peroxide, oxidative stress, cytotoxicity

## Abstract

D-amino acid oxidase (DAAO) is an enzyme that catalyzes the oxidation of D-amino acids generating H_2_O_2_. The enzymatic chimera formed by DAAO bound to the choline-binding domain of N-acetylmuramoyl-L-alanine amidase (CLytA) induces cytotoxicity in several pancreatic and colorectal carcinoma and glioblastoma cell models. In the current work, we determined whether the effect of CLytA-DAAO immobilized in magnetic nanoparticles, gold nanoparticles, and alginate capsules offered some advantages as compared to the free CLytA-DAAO. Results indicate that the immobilization of CLytA-DAAO in magnetic nanoparticles increases the stability of the enzyme, extending its time of action. Besides, we compared the effect induced by CLytA-DAAO with the direct addition of hydrogen peroxide, demonstrating that the progressive generation of reactive oxygen species by CLytA-DAAO is more effective in inducing cytotoxicity than the direct addition of H_2_O_2_. Furthermore, a pilot study has been initiated in biopsies obtained from pancreatic and colorectal carcinoma and glioblastoma patients to evaluate the expression of the main genes involved in resistance to CLytA-DAAO cytotoxicity. Based on our findings, we propose that CLytA-DAAO immobilized in magnetic nanoparticles could be effective in a high percentage of patients and, therefore, be used as an anti-cancer therapy for pancreatic and colorectal carcinoma and glioblastoma.

## 1. Introduction

Increasing knowledge of protein immobilization systems has provided the opportunity to increase the usage of enzymes to several areas and expand it to multiple biotechnological processes, since immobilization increases the half-life and reduces protein degradation [1]. The nature of the immobilization material used plays a fundamental role and determines whether the immobilized protein can reach its full therapeutic potential. The immobilization matrix should be made of affordable and available materials and should provide a wide surface area and a high density of functional groups to facilitate enzyme immobilization. In addition, they should guarantee an adequate diffusion of the substrates and products involved in the enzymatic reaction [2].

Nanoparticles are excellent support materials for enzyme immobilization since, in many cases, they produced an increase in catalytic efficiency. Enzyme immobilization in nanoparticles reduces the limitations in the diffusion of substrates and products and increases the functional surface area and the loading capacity [3], which has a direct impact on the activity of the immobilized enzyme. These characteristics, along with their low toxicity and high biocompatibility and biodegradability, have resulted in the increased use of nanoparticles in biomedical research like in diagnostic tests, drug delivery systems, personalized enzyme therapies, and tissue regeneration [4,5].

Nanoparticles can be classified according to their synthesizing material into inorganic (nanocapsules, silica nanoparticles, gold nanoparticles, quantum dots, magnetic nanoparticles, etc.) and organic (liposomes, polymersomes, micelles, polymeric nanoparticles, dendrimers, etc.), providing each type of material advantages and disadvantages [6]. Specifically, magnetic nanoparticles (MNPs) can be manipulated and targeted using magnetic fields. Many MNPs have been used for the immobilization of proteins with good results [3]. These particles have diameters of 5–500 nm and can be synthesized with iron oxides, such as Fe_3_O_4_ and γ-Fe_2_O_3_, pure metals such as Fe and Co, and mixtures such as CoPt_3_ and FePt [7].

There are several methods to synthetize MNPs: coprecipitation, thermal decomposition, micellar synthesis, hydrothermal synthesis, and laser pyrolysis [7]. After synthesis, magnetic nuclei are protected and stabilized by coating with layers of other compounds to avoid oxidation and decomposition. Normally, the nanoparticle core can be protected with either organic compounds (such as surfactants and polymers) or inorganic compounds (such as silica, carbon, or metals such as Ag and Au) [7]. These coatings not only protect and stabilize the nanoparticle, but also are very frequently used to functionalize the nanomaterial with groups of interest. These possibilities, together with the easy separation and handling, have facilitated their use in biomedical and biotechnological research. They have been applied to protein immobilization and purification, biofuel cells construction, biosensors development, and environmental remediation. In biomedicine, they have been used in applications such as medical equipment development (magnetic resonance imaging) and as drug and gene carriers [3,7,8,9,10].

CLytA-DAAO is an enzymatic chimera formed by the union of the choline-binding domain of N-acetylmuramoyl-L-alanine amidase (CLytA) from *Streptococcus pneumoniae* with the enzyme D-amino acid oxidase (DAAO) from *Rhodotorula gracilis* [11]. This enzyme oxides D-amino acids and, during the reaction, generates hydrogen peroxide as a by-product [12], which is an important inducer of free radicals. The advantage of DAAO over other stress-generating oxidative enzymes, such as glucose oxidase or xanthine oxidase, is that its substrate, D-amino acids, are practically absent in the organism. Thus, the enzyme is not harmful per se and requires the exogenous addition of D-amino acids to be catalytically active [13,14,15]. Previously, we demonstrated the cytotoxic capacity of the enzymatic chimera CLytA-DAAO activated by D-Alanine (D-Ala) in pancreatic carcinoma, colorectal carcinoma, and glioblastoma cell lines [11]. The therapeutic strategy that we propose is based on targeting the enzyme to the tumor area with an external magnetic field in order to concentrate the nanoparticles in the tumor, and then treating the patients systemically with D-amino acids.

The CLytA domain would allow one to immobilize the enzyme in nanoparticles that contain choline or derivatives such as diethylaminoethanol (DEAE) on the surface. In fact, our magnetic nanoparticles have a magnetite core and a DEAE-functionalized starch shell.

Although we demonstrated the cytotoxicity induced by CLytA-DAAO in many pancreatic and colorectal carcinoma and glioblastoma cellular models, we found two cell lines resistant to cell death induced by this treatment: Hs766T from pancreatic carcinoma and HT-29 from colorectal carcinoma [11]. Hs766T resistance to CLytA-DAAO-induced cell death was found to be related to the high expression of antioxidant proteins such as catalase and nuclear factor erythroid 2-related factor 2 (Nrf2) and proteins involved in the inflammatory and stress response such as nuclear factor kappa-light-chain-enhancer of activated B cells (NF-κB) and p38 mitogen-activated protein kinase 11 (p38). Regarding HT-29 resistance to CLytA-DAAO-induced cell death, it is mediated by glutathione peroxidase 2 (GPX2), an antioxidant protein, but also by NF-κB and p38 [16].

The current article evaluates the possible benefits of CLytA-DAAO immobilization in MNPs, focused on demonstration that the cytotoxic capacities of CLytA-DAAO and D-Ala treatment are determined by the progressive induction of oxidative stress. Furthermore, in order to estimate the putative effectiveness of CLytA-DAAO and D-Ala as antitumor treatment in patients, a pilot study has been initiated to find out the general expression of the main genes involved in resistance to CLytA-DAAO in biopsies obtained from patients with pancreatic carcinoma, colorectal carcinoma or glioblastoma.

## 2. Results

### 2.1. The Cytotoxic Effect of CLytA-DAAO Is Higher When It Is Bound to Magnetic Nanoparticles than When It Is Immobilized in Gold Nanoparticles or Alginate Capsules

**Figure 1:**
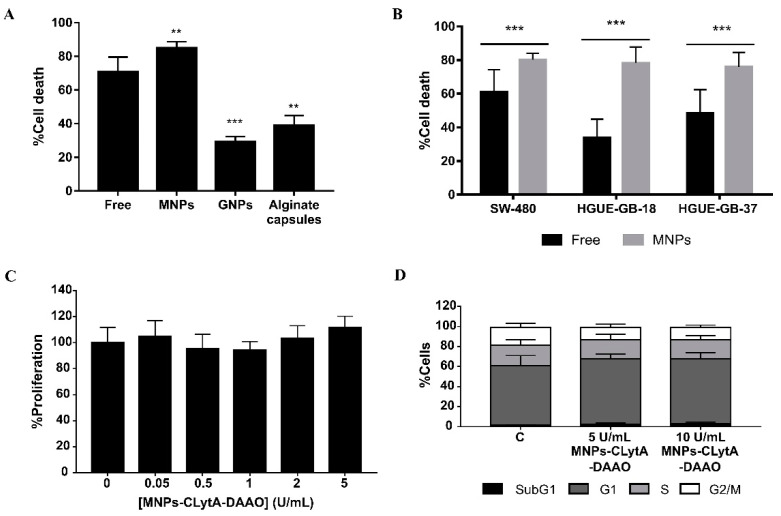
Cell death induced by CLytA-DAAO free, immobilized in magnetic nanoparticles (MNPs), in gold nanoparticles (GNPs), and immobilized in alginate capsules. (**A**) IMIM-PC-2 pancreatic carcinoma cell line was treated with 2 U/mL CLytA-DAAO, free or immobilized in MNPs, GNPs or alginate capsules, and 1 mM D-Ala for 24 h. (**B**) SW-480 colorectal carcinoma cell line and HGUE-GB-18 and HGUE-GB-37 glioblastoma cell lines were treated with 2 U/mL CLytA-DAAO, free or immobilized in MNPs, and 1 mM D-Ala for 24 h. Cell viability was determined using Muse cell analyzer. Graphs represent cell death percentage (mean ± SD) after subtracting cell death in the control untreated (*n* ≥ 3). (**C**) IMIM-PC-2 cell line was treated with CLytA-DAAO bound to MNPs in a concentration range between 0.05–5 U/mL for 72 h. Graph shows the proliferation percentage ± SD normalized with respect to the control untreated (*n* ≥ 6). (**D**) IMIM-PC-2 cell line was treated with 5 or 10 U/mL CLytA-DAAO immobilized in magnetic nanoparticles for 24 h and the cell distribution in each phase of cell cycle was determined by flow cytometry. Graph shows the cell percentage in each phase of cell cycle (mean ± SD) (*n* ≥ 3). ** Indicates a *p*-value < 0.01 and *** *p*-value < 0.001.

In order to compare the CLytA-DAAO effect, either free or immobilized in different immobilization supports, the percentage of cell death produced by the different treatments was determined in the IMIM-PC-2 pancreatic carcinoma cell line, a well-established CLytA-DAAO sensitive cellular model. The enzyme was used free, immobilized in MNPs, in gold nanoparticles (GNPs), or in alginate capsules. A significantly higher percentage of cell death was observed with CLytA-DAAO bound to MNPs in comparison with the free enzyme. However, immobilization in GNPs and in alginate capsules significantly decreased the cell death induced by the treatment (Figure 1A), suggesting that these methods of CLytA-DAAO immobilization are less effective stabilizing the chimeric enzyme than MNPs. We had previously shown that the CLytA-DAAO-bound to MNPs together with D-Ala treatment induces a higher percentage of cells in subG1 phase than the free enzyme in several pancreatic carcinoma, colorectal carcinoma, and glioblastoma cell lines [11]. Here, a series of cell viability analyses were performed to confirm that, in fact, CLytA-DAAO bound to MNPs induced a significantly higher percentage of cell death compared to the free enzyme in all cancer models tested. Figure 1B shows a representative image in SW-480 colorectal carcinoma cell line and in HGUE-GB-18 and HGUE-GB-37 glioblastoma cell lines.

To demonstrate that the higher cell death effect was due to a greater effectiveness of the treatment and not to a possible toxicity derived from MNPs, cell proliferation assays (Figure 1C) and cell cycle analysis (Figure 1D) were performed. For these experiments, we used CLytA-DAAO immobilized in MNPs, in the absence of D-Ala, the substrate of the enzyme. Both assays were carried out on IMIM-PC-2 cell line, and no significant changes were observed with respect to the control. These data demonstrate that MNPs are not toxic per se and, therefore, the greater cell death observed with the enzyme immobilized in MNPs with respect to that observed with free CLytA-DAAO (Figure 1A,B) is due to MNPs immobilization. Taking together these results indicates that CLytA-DAAO immobilized in MNPs improves the effectiveness of the treatment.

### 2.2. The IC50 Value for the Enzyme Immobilized in MNPs Is Lower than for the Free Enzyme

**Figure 2:**
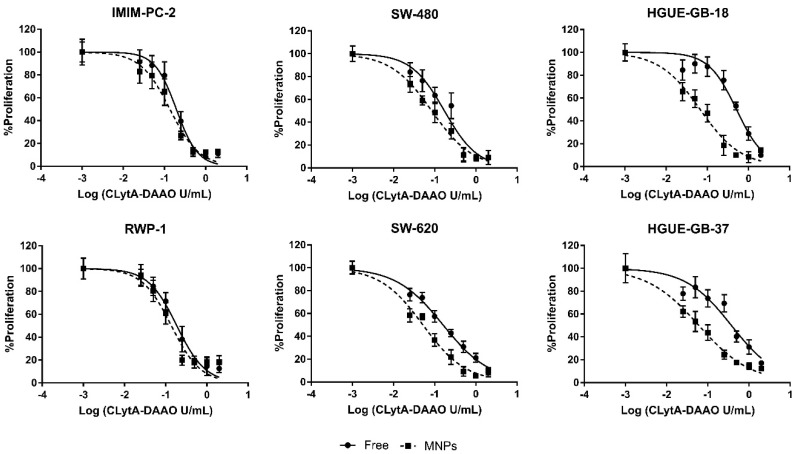
Anti-proliferative effect of CLytA-DAAO, free and bound to MNPs, on pancreatic carcinoma, colorectal carcinoma, and glioblastoma cell lines. Cells were treated with CLytA-DAAO, free or immobilized, in a concentration range between 0.025-2 U/mL and 1 mM D-Ala for 72 h and cell proliferation was determined by MTT assay. Graph shows the proliferation percentage ± SD respect to control untreated versus the logarithm of the concentration (*n* ≥ 6).

To obtain IC50 values of CLytA-DAAO, free, and bound to MNPs, MTT assays were performed. Figure 2 shows the results obtained after 72 h treatment in IMIM-PC-2 and RWP-1 pancreatic carcinoma cell lines, SW-480 and SW-620 colorectal carcinoma cell lines, and HGUE-GB-18 and HGUE-GB-37 glioblastoma cell lines. In all cell lines, a stronger effect was generally observed with CLytA-DAAO bound to MNPs in comparison to free enzyme, although the differences were more evident in glioblastoma cell lines than in pancreatic and colorectal carcinoma cell lines.

Table 1 shows IC50 values obtained with the enzyme, either free or immobilized in MNPs, in several pancreatic carcinoma, colorectal carcinoma, and glioblastoma cell lines. In addition, for a better visualization of the results, the anti-proliferative percentages obtained using 0.025 and 0.25 U/mL of free and immobilized CLytA-DAAO are included.

**Figure 3:**
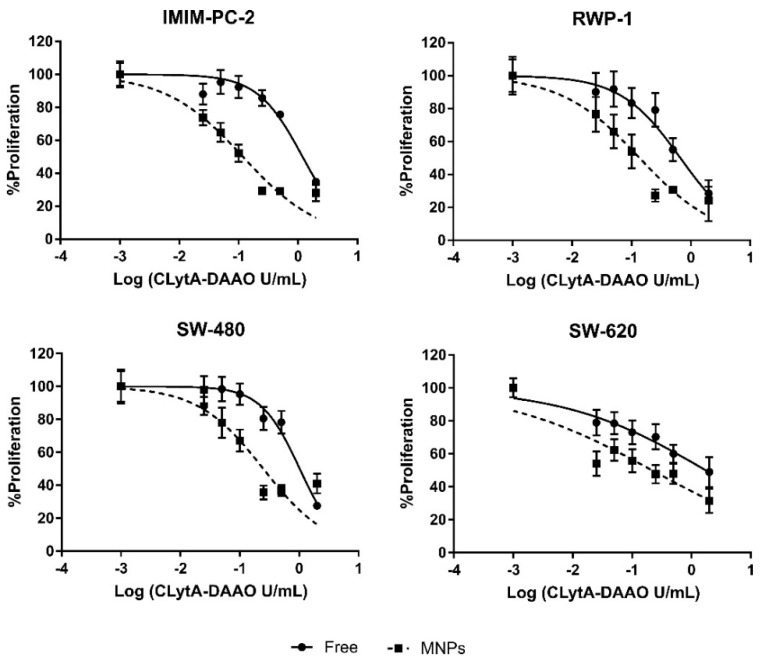
Anti-proliferative effect of CLytA-DAAO, free and bound to MNPs, on pancreatic and colorectal carcinoma cell lines. Cells were treated with CLytA-DAAO, free or immobilized, in a concentration range between 0.025-2 U/mL and 1 mM D-Ala for 24 h and cell proliferation was determined by MTT assay. Graph shows the proliferation percentage ± SD respect to control untreated versus the logarithm of the concentration (*n* ≥ 6).

The differences observed between free and immobilized in MNPs enzyme in viability (Figure 1A,B) and cell cycle analysis [11], were more pronounced than those observed in the MTT assays in pancreatic and colorectal carcinoma cell lines. The viability and cell cycle studies were conducted after 24 h treatment, whereas the MTT assays were carried out after 72 h treatment. To match these conditions, the MTT assays were repeated with a CLytA-DAAO, either free or bound to MNPs, and D-Ala treatment for 24 h in pancreatic carcinoma (IMIM-PC-2 and RWP-1) and in colorectal carcinoma (SW-480 and SW-620 cell lines). It was observed that reducing the treatment time to 24 h, the difference between using the enzyme, free or immobilized in MNPs get increased (Figure 3). The IC50 values obtained with CLytA-DAAO, either free or bound to MNPs, are shown in Table 2.

### 2.3. The Greater Effect Induced by the Treatment with CLytA-DAAO Bound to MNPs Is Due to an Increase in the Enzyme Stability

The cell death induced by CLytA-DAAO and D-Ala treatment is due to the H_2_O_2_ production during the oxidation reaction catalyzed by CLytA-DAAO. In fact, we have previously reported that during the first two hours of treatment, intracellular reactive oxygen species (ROS) levels increased in all cell lines sensitive to CLytA-DAAO-induced cell death [11].

**Figure 4:**
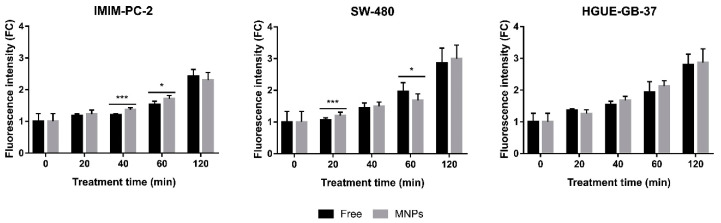
Intracellular reactive oxygen species (ROS) increase after CLytA-DAAO, free and bound to MNPs, and D-Ala treatment in IMIM-PC-2 pancreatic carcinoma cell line, SW-480 colorectal carcinoma cell line, and HGUE-GB-37 glioblastoma cell line. Cells were treated with 2 U/mL CLytA-DAAO and 1 mM D-Ala for 20–120 min. Free radical production was determined through DCFH_2_-DA probe and each treatment time had a control untreated that only contained the probe. Graph shows the fold change (FC) ± SD of fluorescent intensity with respect to the control (*n* ≥ 6). * Indicates a *p*-value < 0.05 and *** < 0.001.

ROS are responsible for the cell death induced by CLytA-DAAO treatment. To evaluate whether the enzyme bound to MNPs is able to increase free radical levels, we used IMIM-PC-2 pancreatic carcinoma cell line, SW-480 colorectal carcinoma cell line, and HGUE-GB-37 glioblastoma cell line. Cells were treated for a time range of 20–120 min with 2 U/mL CLytA-DAAO, either free or immobilized in MNPs, together with 1 mM D-Ala, and the ROS levels were determined with the fluorescent probe DCFH2-DA. In the three cell lines studied, an increase in free radicals was observed depending on the treatment time, without major differences when using free of immobilized CLytA-DAAO (Figure 4). Consequently, the level of ROS reached after each treatment is not the reason that accounted for the difference between cell death levels observed using free or immobilized enzyme.

To determine whether the increase in cell death produced by the immobilized enzyme as compare to that produced by the free enzyme was due to a faster effect of CLytA-DAAO bound to MNPs than of free CLytA-DAAO, the IMIM-PC-2 pancreatic carcinoma, SW-480 colorectal carcinoma, and HGUE-GB-18 glioblastoma cell lines were analyzed. The three cell models were treated with 2 U/mL CLytA-DAO, either free or immobilized in MNPs, and 1 mM D-Ala for different times between 15 and 60 min. Then, the culture medium was replaced, withdrawing the treatment, and the cells were incubated for 24 h to analyze the cell cycle distribution by flow cytometry.

**Figure 5:**
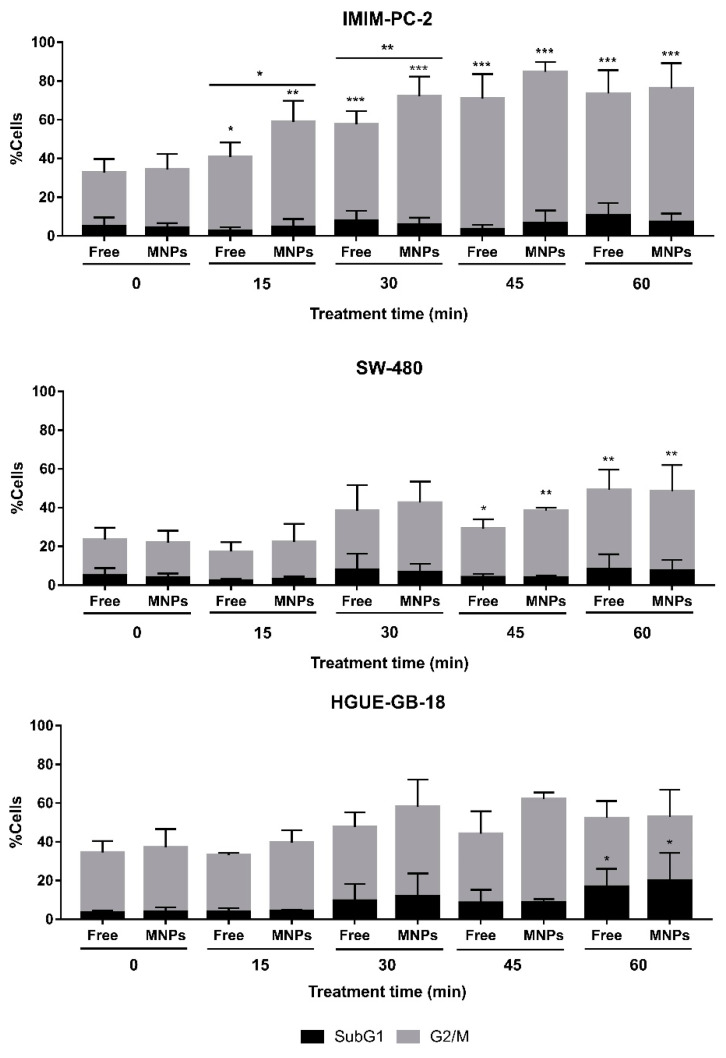
Cells accumulation in subG1 and G2/M phases after a CLytA-DAAO, free or bound to MNPs, and D-Ala treatment in IMIM-PC-2 pancreatic carcinoma cell line, SW-480 colorectal carcinoma cell line, and HGUE-GB-18 glioblastoma cell line. Cells were treated with 2 U/mL CLytA-DAAO and 1 mM D-Ala for a short time (15–60 min) and then, treatment was removed, replacing the medium. Cells were incubated until 24 h from the treatment addition were completed. Graph shows the percentage of cells ± SD in subG1 and G2/M phases (*n* ≥ 3). * Indicates a *p*-value < 0.05, ** < 0.01 and *** < 0.001.

It should be noted that in pancreatic and colorectal carcinoma cell lines, an initial cytostatic effect of the enzyme was observed, blocking the cells in the G2/M phase, while in the glioblastoma cell line, the effect was exclusively cytotoxic (Figure 5). This result agrees with previous data showing that CLytA-DAAO induces cell death by a different mechanism depending on the tumor model. A traditional apoptosis was described in glioblastoma cell lines, whereas pancreatic and colorectal carcinoma cell lines displayed a necrotic-like cell death [11,16]. The results showed slight changes between the immobilized and free CLytA-DAAO, suggesting that CLytA-DAAO bound to MNPs acts faster than free CLytA-DAAO. However, the differences were not statistically significant in most cases (Figure 5). The same effects were observed in other pancreatic and colorectal carcinoma cell lines (Supplementary Materials Figure S1).

**Figure 6:**
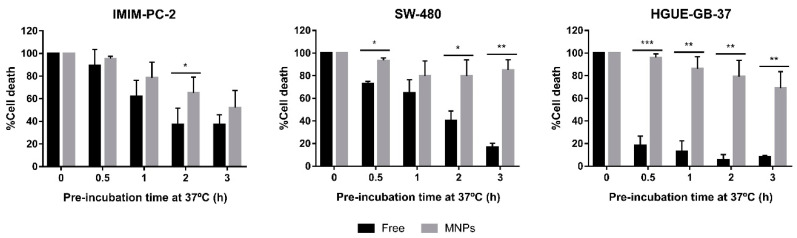
Cell death induced by CLytA-DAAO, free and bound to MNPs, and D-Ala after incubation of the enzyme at 37 °C in IMIM-PC-2 pancreatic carcinoma cell line, SW-480 colorectal carcinoma cell line, and HGUE-GB-37 glioblastoma cell line. Cells were treated with 2 U/mL CLytA-DAAO and 1 mM D-Ala for 24 h. The CLytA-DAAO used was pre-incubated at 37 °C for 30 min, 1, 2, and 3 h before adding it to the cells. Graph shows the percentage of cell death normalizing the treatment with CLytA-DAAO without pre-incubation as 100% ± SD (*n* ≥ 3). * Indicates a *p*-value < 0.05, ** < 0.01 and *** < 0.001.

Finally, since CLytA-DAAO is a yeast enzyme and its optimum temperature is 25 °C, the stability of the enzyme at 37 °C was determined in IMIM-PC-2 pancreatic carcinoma, SW-480 colorectal carcinoma, and HGUE-GB-37 glioblastoma cell lines. To analyze the stability, CLytA-DAAO either free or immobilized in MNPs was incubated at 37 °C for 0.5, 1, 2, and 3 h before being added to the cells with D-Ala. Then, the treatment was maintained for 24 h and cell viability was studied. Results show statistically significant differences between the two conditions (Figure 6). CLytA-DAAO bound to MNPs maintained the cell death effect for a longer time than in its free state, which indicates that the immobilization in MNPs substantially improves the stability of the enzyme at 37 °C.

### 2.4. CLytA-DAAO Can Be Released from MNPs Through the External Addition of Choline

An advantage of our system is that CLytA-DAAO immobilization in MNPs is carried out by the affinity of the CLytA domain for choline and derivates such as DEAE, which is used in this case to functionalize the MNPs [17,18]. The main advantage of this type of immobilization is that the process is very simple and reversible.

**Figure 7:**
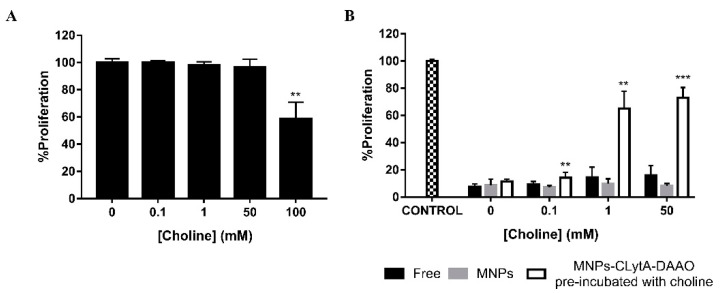
CLytA-DAAO release from MNPs through the choline addition. (**A**) SW-480 cell line was treated with 1–100 mM choline for 72 h and cell proliferation was determined by MTT assay. (**B**) SW-480 cell line was treated with 2 U/mL CLytA-DAAO, 1 mM D-Ala and increasing concentrations of choline (0.1–50 mM) for 72 h and cell proliferation was determined by MTT assay. The CLytA-DAAO enzyme was used: free and bound to MNPs in combination with choline and bound to MNPs after a pre-incubation with choline for 10 min. Graphs show the proliferation percentage ± SD respect to control untreated (*n* ≥ 6). ** Indicates a *p*-value < 0.01 and *** < 0.001.

To evaluate the possibility of releasing the enzyme from MNPs, MTT assays were initially performed to verify the toxicity of choline. Increasing doses of choline were added to the SW-480 colorectal carcinoma cell line for 72 h, and only the 100 mM concentration decreased cell proliferation (Figure 7A). Next, additional MTT assays were carried out combining CLytA-DAAO, D-Ala and choline under different conditions. 2 U/mL CLytA-DAAO, either free or bound to MNPs, were added in combination with 1 mM D-Ala and different concentrations of choline (0.1, 1, and 50 mM). In addition, CLytA-DAAO bound to MNPs was pre-incubated with choline (0.1, 1, and 50 mM) for 10 min and the released CLytA-DAAO was removed with the aid of an external magnet. The enzyme that remained immobilized in the MNPs was added to cells. CLytA-DAAO, either free or immobilized in MNPs, maintained its maximum effect with the different doses of choline added (Figure 7B). On the other side, treatment of cells with the enzyme that had been pre-incubated with choline, shows a significant decrease in the anti-proliferative effect dependent on the dose of choline was observed (Figure 7B).

### 2.5. CLytA-DAAO and D-Ala Induce Cell Death in a Different Way than Direct H_2_O_2_ Addition

**Figure 8:**
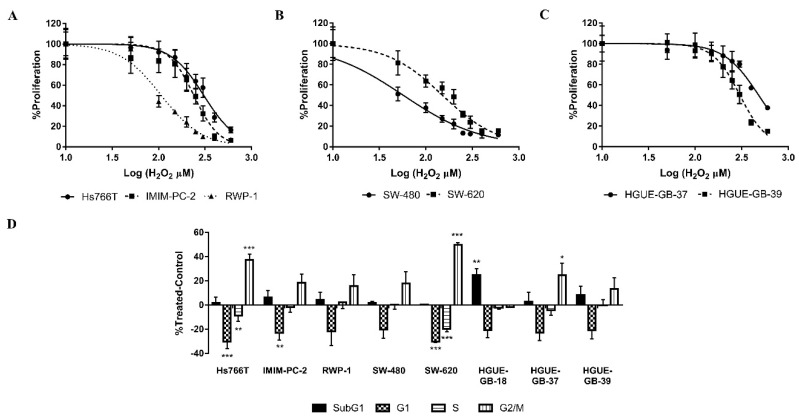
H_2_O_2_ effect in pancreatic carcinoma, colorectal carcinoma, and glioblastoma cell models. H_2_O_2_ anti-proliferative effect induced in pancreatic carcinoma (**A**), colorectal carcinoma (**B**) and glioblastoma (**C**) cell lines. Cells were treated with H_2_O_2_, in a concentration range between 50–600 μM for 72 h and cell proliferation was determined by MTT assay. Graph shows the proliferation percentage ± SD respect to control untreated versus the logarithm of the concentration (*n* ≥ 6). (**D**) Variations in cell cycle distribution after H_2_O_2_ treatment in Hs766T, IMIM-PC-2, and RWP-1 pancreatic carcinoma cell lines, SW-480 and SW-620 colorectal carcinoma cell lines and HGUE-GB-18, HGUE-GB-37, and HGUE-GB-39 glioblastoma cell lines. Cells were treated with 600 μM H_2_O_2_ for 24 h and cell cycle distribution was determined by flow cytometry. Graph shows the cells percentage ± SD in each phase of cell cycle after subtracting the cells percentage in the control untreated (*n* ≥ 3). * Indicates a *p*-value < 0.05, ** < 0.01 and *** < 0.001.

As mentioned above, cell death induced by CLytA-DAAO and D-Ala is supposed to be caused by the H_2_O_2_ production, which leads to increased ROS levels within the cells causing death. In order to check whether CLytA-DAAO and D-Ala treatment are equivalent to add directly H_2_O_2_ to cells, MTT assays were performed. We used increasing doses of H_2_O_2_ in a range between 50 and 600 μM in pancreatic, colorectal, and glioblastoma tumor cell lines. A concentration-dependent anti-proliferative effect in all cell lines tested can be seen (Figure 8A–C). Table 3 shows the IC50 values for the H_2_O_2_ treatment. Results showed different sensitivity patterns compared to CLytA-DAAO and D-Ala treatment. IMIM-PC-2 and RWP-1 pancreatic carcinoma cell lines, as well as SW-480 and SW-620 colorectal carcinoma cell lines showed similar sensitivity levels under CLytA-DAAO and D-Ala treatment. However, IMIM-PC-2 and SW-620 seem to be more resistant to H_2_O_2_ treatment than RWP-1 and SW-480. Finally, both glioblastoma cell lines appear to be quite resistant to H_2_O_2_ treatment, with HGUE-GB-37 cell line being the most resistant of all cell lines.

Next, cell cycle analyses were performed after 600 μM H_2_O_2_ treatment for 24 h to study the cell death effect. Results showed important differences with respect to the treatment with CLytA-DAAO and D-Ala. In IMIM-PC-2 and RWP-1 pancreatic carcinoma cell lines as well as in SW-480 and SW-620 colorectal carcinoma cell lines, the treatment with 2 U/mL CLytA-DAAO and 1 mM D-Ala produces a markedly cytotoxic effect (data not shown). In contrast, in IMIM-PC-2, RWP-1 and SW-480 cell lines, treatment with H_2_O_2_ induced a lighter effect as compared to the observed with CLytA-DAAO, showing a small increase in the percentage of cells in subG1 and G2/M phases with respect to the control (Figure 8D). Curiously, SW-620 did not show an increase in the percentage of dead cells, but the effect caused by H_2_O_2_ was cytostatic, increasing the percentage of cells in the G2/M phase to 49.6 ± 2.12% with respect to the control (Figure 8D). Hs766T pancreatic carcinoma cell line was resistant to cell death induced by CLytA-DAAO and D-Ala, with an increase in subG1 phase of 6.81 ± 5.21% (data not shown). However, a pronounced increase in the percentage of cells in G2/M phase was observed after H_2_O_2_ treatment (Figure 8D). Finally, in glioblastoma cell models the changes in cell cycle distribution were also lower than the observed with CLytA-DAAO and D-Ala treatment. Only HGUE-GB-18 showed an increase in the subG1 phase very similar to that observed with CLytA-DAAO in previous studies (Figure 8D).

**Figure 9:**
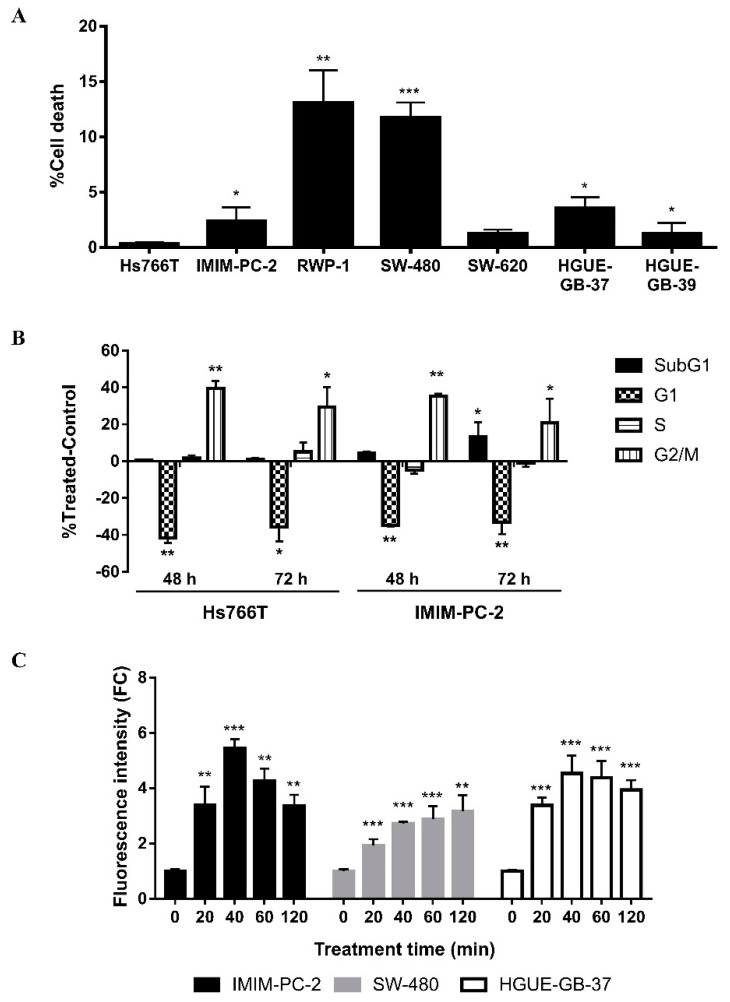
Differential effect of H_2_O_2_ with respect to CLytA-DAAO and D-Ala treatment. (**A**) Plasmatic membrane rupture induced by H_2_O_2_ in Hs766T, IMIM-PC-2, and RWP-1 cell lines from pancreatic carcinoma, SW-480 and SW-620 cell lines from colorectal carcinoma and HGUE-GB-37 and HGUE-GB-39 cell lines from glioblastoma. Cells were treated with 600 μM H_2_O_2_ for 24 h and cell viability was determined using Muse cell analyzer. Graph represents cell death percentage (mean ± SD) after subtracting cell death in the control untreated (*n* ≥ 3). (**B**) Variations in cell cycle distribution after H_2_O_2_ treatment for 48 and 72 h. Hs766T and IMIM-PC-2 pancreatic carcinoma cell lines were treated with 600 μM H_2_O_2_ for 48 and 72 h and cell cycle distribution was determined by flow cytometry. Graph shows the cells percentage ± SD in each phase of cell cycle after subtracting the cells percentage in the control untreated (*n* ≥ 3). (**C**) Intracellular ROS increase after H_2_O_2_ treatment in IMIM-PC-2 pancreatic carcinoma cell line, SW-480 colorectal carcinoma cell line and HGUE-GB-37 glioblastoma cell line. Cells were treated with 600 μM H_2_O_2_ for 20–120 min. Free radical production was determined through DCFH_2_-DA probe and each treatment time had a control untreated that only contained the probe. Graph shows the fold change (FC) ± SD of fluorescent intensity with respect to the control (*n* ≥ 6). * Indicates a *p*-value < 0.05, ** *p*-value < 0.01 and *** *p*-value < 0.001.

These analyses were repeated, evaluating the percentage of cell death through the plasmatic membrane rupture by a viability assay. In all cell lines, the percentage of cell death was less than 15%, although RWP-1 pancreatic carcinoma cells and SW-480 colorectal carcinoma cells suffered a higher percentage of cell death (Figure 9A). Therefore, with H_2_O_2_ treatment, the percentage of cell death did not reach the levels observed with CLytA-DAAO and D-Ala treatment in any of the cell lines tested.

One of the main questions raised by these findings is whether the G2/M blockade observed in Hs766T eventually turned into cell death. To address this, cell cycle analyses were repeated using 48 and 72 h treatment. In addition, we included IMIM-PC-2 cell line to find out whether a longer treatment was necessary to obtain similar results to those observed with CLytA-DAAO and D-Ala treatment. Hs766T maintained the G2/M phase blockade up to 72 h of H_2_O_2_ treatment (Figure 9B). On the contrary, 72 h of treatment with H_2_O_2_ were enough to observe a significant increase in the percentage of cells in subG1 phase, corresponding to dead cells, in the IMIM-PC-2 cell line (Figure 9B). However, none of these conditions reached the level of cytotoxicity generated by CLytA-DAAO and D-Ala treatment.

Cell death induced by CLytA-DAAO is due to a progressive increase in the ROS levels inside the cells. Therefore, ROS produced by the treatment with H_2_O_2_ were determined in IMIM-PC-2 cell line. After two hours of treatment with CLytA-DAAO, ROS levels increased 2.43 ± 0.21 times with respect to the control (Figure 4), and 40 min after H_2_O_2_ addition, the maximum ROS levels were reached (5.45 ± 0.32) with a subsequent decrease (Figure 9C). This observation indicates that addition of CLytA-DAAO and D-Ala results in a progressive increase of ROS, but the time conditions used in this treatment are not optimal to observe the maximum effect. Instead, H_2_O_2_ treatment leads to faster ROS levels increase that is followed by a decrease. In the HGUE-GB-37 cell line, these decreases in ROS levels were also observed after two hours of H_2_O_2_ treatment (Figure 9C). Surprisingly, two hours of treatment in SW-480 were not enough to observe the decrease of ROS (Figure 9C). In conclusion, cell death induced by CLytA-DAAO and D-Ala is due to a progressive and sustained in time increase in ROS levels. The differential effects observed between H_2_O_2_ and CLytA-DAAO treatment are graphically summarized in Supplementary Materials Figure S2.

### 2.6. Expression of Genes Involved in Resistance to CLytA-DAAO-Induced Cell Death in Patient Samples

The resistance mechanisms against CLytA-DAAO-induced cell death in Hs766T cell line from pancreatic carcinoma and in HT-29 cell line from colorectal carcinoma were determined in our previously published article. Resistance mechanisms were related to the expression of some genes involved in the response to oxidative stress and cell survival [16].

The expression of three of the main genes involved in the resistance to CLytA-DAAO treatment was analyzed in biopsies from patients with pancreatic carcinoma, colorectal carcinoma, and glioblastoma. However, given the reduced number of biopsies obtained until now, we have complemented the analysis using UALCAN database. This web portal allows analysis of gene expression and survival in different types of tumors using data extracted from “the Cancer Genome Atlas” project [19].

**Figure 10:**
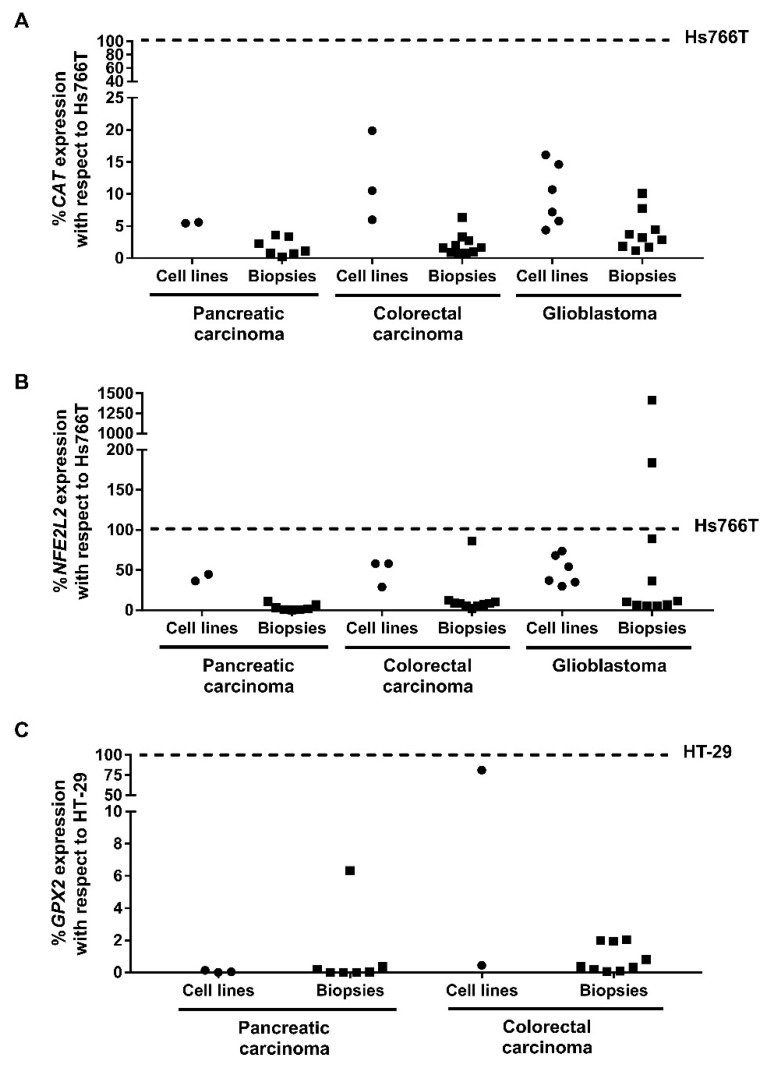
Gene expression in cell lines and patient biopsies from pancreatic carcinoma, colorectal carcinoma, and glioblastoma. Graphs show the expression percentage of *CAT* (**A**), *NFE2L2* (**B**), and *GPX2* (**C**). *CAT* and *NFE2L2* expression are normalized with respect to the expression observed in Hs766T pancreatic carcinoma cell line and *GPX2* is normalized with the expression observed in HT-29 colorectal carcinoma cell line (*n* ≥ 3).

Catalase (*CAT*) is one of the main genes over-expressed in Hs766T and involved in resistance against CLytA-DAAO-induced cell death. *CAT* expression in the rest of pancreatic carcinoma (IMIM-PC-2 and RWP-1), colorectal carcinoma (HT-29, SW-480, and SW-620) and glioblastoma (HGUE-GB-18, HGUE-GB-37, HGUE-GB-39, and HGUE-GB-42) cell lines were more than 80% lower than that observed in Hs766T (Figure 10A). Besides, Figure 10A shows the *CAT* expression in patient biopsies: in pancreatic carcinoma biopsies is in the range of 0.22%–3.62% with respect to that observed in Hs766T, in colorectal carcinoma between 0.79%–6.35% and in glioblastoma between 1.14%–10.1%. This expression levels were more similar to the observed in sensitive cell lines ranged between 5.5%–20% with respect to the CAT expression in Hs766T (Figure 10A). In the UALCAN analysis, a similar *CAT* expression among pancreatic carcinoma, colorectal carcinoma, and glioblastoma patients was observed (Supplementary Materials Figure S3A). Only, in patients with colon adenocarcinoma, *CAT* expression is significantly lower than in normal tissue (Supplementary Materials Figure S3A), although this does not seem to affect survival [19].

Another gen involved in the resistance of Hs766T to cell death induced by CLytA-DAAO is *NFE2L2*, which encodes the Nrf2 transcription factor. The *NFE2L2* expression in pancreatic carcinoma, colorectal carcinoma, and glioblastoma cell lines was between 29%–73.7% with respect to the observed in Hs766T (Figure 10B). In the patient biopsies, the *NFE2L2* expression was highly variable. Biopsies from pancreatic carcinoma patients maintained an expression lower than 15% with respect to the expression observed in Hs766T. A similar result was obtained in colorectal carcinoma patients, with one exception, where *NFE2L2* expression was 61.36% ± 13.2% with respect to Hs766T expression (Figure 10B). NFE2L2 expression in pancreatic and colorectal carcinoma cell lines was comprised between 29%–58% with respect to the observed in Hs766T (Figure 10B), thus the average percentage of expression in patients is significantly lower than the observed in sensitive cell lines. However, in glioblastoma biopsies, six samples showed an expression lower than 15%, three of them had 36.41% ± 14.66%, 88.95% ± 16.27%, and 183.74% ± 19.4%, and there was one biopsy whose expression was 1246.81% ± 171.47% with respect to Hs766T (Figure 10B). In the glioblastoma cell lines, the NFE2L2 expression levels ranged between 30%–73.7% with respect to the observed in Hs766T (Figure 10B).

Survival analysis performed with UALCAN showed that a high *NFE2L2* expression in pancreatic carcinoma patients has a correlation with poor prognosis (*p*-value: 0.023), while in colorectal carcinoma and glioblastoma patients, no significant changes were observed in survival analysis (data not shown). On the other hand, colorectal carcinoma patients presented a lower *NFE2L2* expression with respect to the healthy tissue, while glioblastoma patients have a higher expression in comparison with healthy brain tissue samples (Supplementary Materials Figure S3B). However, similar to *CAT*, there were no differences in expression between pancreatic carcinoma, colorectal carcinoma, and glioblastoma patients (Supplementary Materials Figure S3B).

A high expression of *GPX2* was related with the cell death resistance observed in HT-29 colorectal carcinoma cell line. *GPX2* expression in pancreatic carcinoma cell lines was in the range 0.005–0.120% with respect to HT-29 expression (Figure 10C). However, in glioblastoma cell lines, there was no expression of *GPX2*. Regarding the expression obtained in biopsies, six of the pancreatic carcinoma patients and six of the colorectal carcinoma patients showed expression levels similar to those observed in pancreatic carcinoma cell lines and in SW-480 colorectal carcinoma cell line, lower than 0.5% with respect to the expression in HT-29 (Figure 10C). The highest expression was observed in one of the pancreatic carcinoma biopsies, with 6.32% ± 0.79% with respect to HT-29 and, the remaining four colorectal carcinoma biopsies were between 0.8%–2.03% (Figure 10C). In contrast, no *GPX2* expression was observed in any of the glioblastoma biopsies.

In the analysis performed in UALCAN, the highest *GPX2* expression occurred in colorectal carcinoma patients, followed by pancreatic carcinoma patients, in which *GPX2* expression is approximately 85% with respect to the expression observed in colorectal carcinoma. In glioblastoma patients, the expression was around 0.6% with respect to the observed in colorectal carcinoma (Supplementary Materials Figure S3C). The expression in colorectal carcinoma patients was higher than the expression observed in healthy tissue (Supplementary Materials Figure S3C). Furthermore, Kaplan–Meier analysis suggests that a high *GPX2* expression in glioblastoma patients results in a low survival rate (*p*-value: 0.0035).

## 3. Discussion

DAAO has important biotechnological applications such as biosensor, resistance mechanism to herbicides, bioreactor in the synthesis of semisynthetic cephalosporins or α-ketoacids, or in biomedicine, since it has been shown to present cytotoxicity against various tumor cell models [20]. Recently, we demonstrated the cytotoxic effect induced by DAAO with D-Ala in several cell lines from pancreatic carcinoma, colorectal carcinoma, and glioblastoma with minimal effects on non-tumor cells [11]. However, free DAAO is unstable. Thus, its immobilization on different support materials and through several strategies has been evaluated in order to increase its stability against pH, hydrogen peroxide or temperature [21,22,23,24,25,26]. DAAO immobilization in MNPs has been achieved both by covalent methods [27,28] and by affinity interactions through histidine tag [26,29] or the avidin/streptavidin system [30]. In the current study, DAAO protein has been linked to the CLytA domain (CLytA-DAAO), which provides a high affinity for choline and derivatives, allowing its immobilization in nanoparticles by affinity methods. This fact has major implications since it has been shown that nanoparticles can cross biological barriers such as the blood–brain barrier, facilitating the access of drugs to brain tumors [31,32].

The main challenge related to the immobilization of enzymes in nanoparticles is to get the protein to bind in a stable way to the immobilization support, retaining most of its biological activity. Among immobilization strategies, non-covalent methods have important advantages: (1) they are gentle procedures; (2) do not require chemical modification of the protein, thus reducing the risk of loss of activity and/or denaturation; (3) in most cases the methods are reversible, which allows the reuse of nanoparticles. Additionally, the use of affinity polypeptides provides the necessary degree of specificity that lack many purely physical adsorption methods by directing the immobilization of the protein through affinity interactions between the tag and the immobilizing support.

In this article, we have compared the immobilization of CLytA-DAAO enzyme in MNPs, GNPs, and sodium alginate capsules, although only the immobilization on MNPs showed a significantly higher effect than that obtained with the free enzyme (Figure 1A,B).

GNPs are able to bind thiol groups and, this property has been extensively used to functionalize GNPs with amino acids and proteins for medical applications and biological activities [33,34,35]. GNPs have optical and electrical properties that make them very interesting. In addition, they have low toxicity, are potentially biodegradable, chemically and physically stable, and easy to functionalize [36,37,38,39]. The optical and electrical properties of GNPs are dependent on surface plasmon resonance (SPR), which involves the fluctuation and interaction of electrons between positive and negative charges on the surface [38,40]. Due to SPR, when GNPs are irradiated at a certain wavelength, they transform light energy into heat, which can cause hyperthermia or thermal ablation [39]. So far, they have been studied as drug carriers [41], in photodynamic therapy [42] and in diagnostics to detect biomarkers of different types of diseases [43]. We have found that they were less effective than MNPs to retain CLytA-DAAO activity, probably because the binding through the gold affinity for thiol groups interferes with CLytA-DAAO conformation modifying its activity, meanwhile than the binding through the CLytA tag did not. However, it may be that CLytA-DAAO immobilized in GNPs combined with the possibility to generate hyperthermia inducing SPR might turn this immobilization of CLytA-DAAO into a useful method for clinical application.

The enzyme has also been immobilized by alginate capsules trapping. Alginate is one of the most widely used polymers in encapsulation due to its high versatility and biocompatibility and because the gel capsules generated protect the immobilized component, increasing its stability and bioavailability [44]. This method has been proven to be less effective retaining CLytA-DAAO activity than MNPs (Figure 1A), maybe because the alginate trap interferes with the interaction between the enzyme and the cells, or because part of the H_2_O_2_ produced is trapped in the alginate and do not reach the cells.

The structural similarity between DEAE and choline allows us to easily immobilize the chimeric enzyme by the affinity of the CLytA tag for DEAE. This type of immobilization is rapid, specific, and reversible through DEAE’s affinity for the CLytA tag. The main advantage of MNPs is the possibility of directing the immobilized drug towards the tumor area through the application of an external magnetic field [45,46]. In addition, they have the advantage that they can be visualized by magnetic resonance imaging [47]. Other interesting feature is that superparamagnetic nanoparticles (<50 nm) produce heat after the application of magnetic fields and therefore can be used for thermal ablation of the tumor [48]. Currently, the FDA has approved Nanotherm^®^ as a treatment against glioblastomas, formed by nanoparticles of iron oxide of 15 nm covered with aminosilane [49,50].

Previously, it was demonstrated that DAAO covalently immobilized in MNPs induces cytotoxicity [28]. In our study, a simpler immobilization method has been used, that only involves incubation with nanoparticles. The MNPs used in the previous study showed a certain degree of cytotoxicity per se, which did not occur in our case (Figure 1C,D), opening the possibility to concentrate the nanoparticles on the tumor, avoiding side effects.

These data demonstrate the promising utility of the immobilization of CLytA-DAAO in MNPs. Therefore, our research has focused on the characterization of the greater cytotoxic effect induced by the immobilized enzyme compared to that generated by free CLytA-DAAO. First, dose-response analyses were carried out in several cell models from exocrine pancreatic carcinoma, colorectal carcinoma, and glioblastoma, which confirmed that the enzyme immobilized in MNPs was more effective than the free enzyme (Figure 2), presenting lower IC50 values (Table 1). This difference was more evident when treatment time was reduced to 24 h. This effect was especially evident in those cell lines in which the cytotoxic effect of the free enzyme was already very pronounced, as in exocrine pancreatic carcinoma and colorectal carcinoma cell lines (Figure 3, Table 2).

The increased effect produced by the immobilized enzyme in MNPs was due to an increase in the stability of the enzyme, i.e., the enzyme immobilized in MNPs maintains its effect for longer than the free CLytA-DAAO (Figure 6). We discarded the possibility that this could be caused by a higher ROS production (Figure 4) or by a greater speed to produce its effect on tumor cell lines (Figure 5). It is well known that the stability of *R. gracilis* DAAO decreases dramatically above 30 °C [51], and the normal temperature of human cells is 37 °C. Along this line, there are several studies describing the thermal stabilization of DAAO by immobilization on different support materials [26,27,30,52]. In turn, pH in the tumor area is lower than in the rest of the organism. It has also been described that the DAAO stability in pH acid increases when it is immobilized on positively charged supports [27], as occurs with the MNPs used in this work, while decreases when it is immobilized on negatively charged supports [26]. Finally, another advantage of the non-covalent immobilization system is the controlled release of the enzyme from MNPs that can be induced once localized in the tumor by external addition of choline (Figure 7).

To turn the CLytA-DAAO/D-Ala system into a potential anti-cancer treatment, it is important to explore the cell death induction mechanism. In previous studies, it was determined that cell death is dependent on the intracellular ROS increase caused by the reaction catalyzed by CLytA-DAAO [11]. These ROS caused DNA damage and plasma membrane rupture [11], as well as a decrease in the mitochondrial membrane potential during the first hours of treatment with CLytA-DAAO [16]. Besides, here we have demonstrated that this effect is not equivalent to the direct administration of H_2_O_2_ on tumor cell lines. The dose-response analyses carried out to determine the H_2_O_2_ concentration equivalent to the CLytA-DAAO dose to induce the maximum effect on tumor cell lines after 72 h of treatment, already showed differences in the effect (Figure 8A–C, Table 3). Some of the cell lines sensitive to cell death induced by CLytA-DAAO, such as IMIM-PC-2 from pancreatic carcinoma and SW-620 from colorectal carcinoma (Figure 2, Table 1), were more resistant to the simple addition of H_2_O_2_ (Figure 8A,B, Table 3). In any case, it was determined that 600 μM H_2_O_2_ induced an anti-proliferative effect similar to that produced by 2 U/mL CLytA-DAAO and 1 mM D-Ala (Figure 8A–C, Table 3).

Consequently, cells were treated with 600 μM H_2_O_2_ to analyze the cell cycle distribution, the plasma membrane rupture, and the intracellular ROS increase during the first hours of treatment. Regarding the cell cycle analysis, the main effect induced by H_2_O_2_ in most cell lines was a blockade in G2/M phase, with the exception of HGUE-GB-18 glioblastoma cell line, in which there was an increase in the subG1 phase (Figure 8D), equivalent to the DNA fragmentation observed with the CLytA-DAAO treatment [11]. The anti-proliferative effect of H_2_O_2_ has been described in lung cancer (Calu-6 and A549) and cervical cancer (HeLa) cell lines with IC50 values of ~50, ~100 and ~75 μM respectively after 24 h of treatment [53,54]. However, both studies showed that only doses below 100 μM produced an increase in the subG1 phase. Moreover, an increase in the percentage of dead cells was observed through trypan blue assays using doses higher than 100 uM [53,54].

The viability assays using 600 μM H_2_O_2_ revealed the rupture of the plasma membrane mainly in RWP-1 pancreatic carcinoma cell line and SW-480 colorectal carcinoma cell line (Figure 9A), although never reaching the values induced by CLytA-DAAO and D-Ala treatment [11]. Even an increase in the treatment time up to 72 h did not produce the cytotoxicity levels induced by CLytA-DAAO (Figure 9B). In conclusion, the effect induced by H_2_O_2_ is mainly cytostatic, while CLytA-DAAO and D-Ala cause a cytotoxic effect. Interestingly, the pancreatic carcinoma cell line Hs766T showed a marked cytostatic effect after the H_2_O_2_ treatment (Figure 8D and Figure 9B) while it is resistant to CLytA-DAAO and D-Ala treatment [11].

The differences observed with H_2_O_2_ treatment with respect to CLytA-DAAO and D-Ala can be explained by the intracellular ROS increase. Generally, H_2_O_2_ causes a higher ROS increase than CLytA-DAAO and D-Ala. However, ROS levels also decrease more rapidly (Figure 4 and Figure 9C). Meanwhile, the enzyme produces ROS continuously until the loss of its activity, so the addition of H_2_O_2_ is not enough to induce a similar cytotoxic effect on tumor cell lines (Supplementary Materials Figure S2 show the H_2_O_2_ and CLytA-DAAO differential effects). However, more analyses are needed to determine whether there is any other factor influencing the cytotoxic effect induced by CLytA-DAAO.

Another factor to consider in order to determine the potential of CLytA-DAAO as an anti-cancer therapy is the percentage of patients who would be sensitive to this treatment. In cell model studies, two cell lines resistant to cell death induced by CLytA-DAAO were mainly detected: Hs766T from exocrine pancreatic carcinoma and HT-29 from colorectal carcinoma. Subsequently, the resistance mechanisms present in both cell lines were studied [16]. The resistance of Hs766T is related to a high antioxidant response dependent on the high expression of *CAT* and *NFE2L2* genes, but also on a high stress response mediated by p38 and NF-κB [16]. In the case of HT-29, the high basal expression of *GPX2*, an antioxidant gene, influences the resistance but, as in Hs766T, the stress response has an important role [16].

Since the antioxidant response seems to be crucial in the resistance against CLytA-DAAO-induced cell death, in the current work, a pilot study has been initiated to evaluate the *CAT*, *NFE2L2* and *GPX2* expression in biopsies from patients with pancreatic carcinoma, colorectal carcinoma, and glioblastoma (Figure 10). The data obtained were complemented with gene expression data extracted from UALCAN database (Supplementary Materials Figure S1).

It is important to highlight that we used the UALCAN database, being aware that our data, obtained in biopsies, are not directly comparable with the data from UALCAN. This is because our data were obtained by q-PCR analysis, and the gene expression was normalized with the expression of an internal control, meanwhile that the UALCAN data were obtained by differential gene expression analysis and are represented as transcripts of a specific gene per million of transcripts. It is also important to point out that UALCAN database always consider the 25% of the patients with higher values as high expression patients and the 75% remaining patients as medium and lower expression patients. The information that we want to extract from UALCAN is basically whether a specific gene is differentially expressed in comparison with the non-tumor tissue in pancreatic and colorectal carcinoma as well as in glioblastoma and, whether the expression of these genes has been related to patient’s survival as an indirect probe that could be involved in antitumoral resistant mechanism.

Taking in consideration only the biopsies of the three types of tumors analyzed, our data found that *CAT* expression, was high in the 43% of pancreatic carcinoma biopsies, in the 40% of colorectal biopsies and in the 60% of glioblastoma samples (Figure 10A). With respect to *NFE2L2*, 10% of patients with pancreatic carcinoma, 60% of patients with colorectal carcinoma and 50% glioblastoma biopsies have high expression levels (Figure 10B). In addition, *GPX2* is overexpressed in 11% and 60% of biopsies with pancreatic and colorectal carcinoma, respectively, while it is not expressed in glioblastoma (Figure 10C). Low or no expression of GPX2 observed in glioblastoma cell lines and biopsies is in accordance with data collected in UALCAN.

The higher variability in *CAT* and *NFE2L2* expression was observed in glioma biopsies, which also had the highest expression levels (Figure 10A,B). Our analysis using the UALCAN database, however, showed that *CAT* expression was more variable in pancreatic and colorectal carcinoma patients, while the *NFE2L2* expression was very similar between the three types of tumors (Supplementary Materials Figure S3A,B). Certainly, glioblastoma patients showed higher expression of *NFE2L2* with respect to normal tissue (Supplementary Materials Figure S3B). In contrast, while *GPX2* expression between pancreatic and colorectal carcinoma biopsies was very similar in our experiments (Figure 10C), UALCAN data indicate higher variability among pancreatic carcinoma patients (Supplementary Materials Figure S3C). Furthermore, the expression in patients with colorectal carcinoma was significantly higher in comparison with normal tissue (Supplementary Materials Figure S3C).

Finally, when comparing the expression obtained in the biopsies with the observed in the resistant cell lines, the expression of *CAT* and *GPX2* was less than 10% in all the biopsies studied, while there was more variability in the case of *NFE2L2* (Figure 10). Thus, pancreatic and colorectal carcinoma biopsies had an expression level of less than 10% with respect to Hs766T cell line, with the exception of one colorectal carcinoma patient who has 40% lower expression with respect to Hs766T (Figure 10B). And, although there was a lot of variability between glioma biopsies, it should be noted that there were two patients who had an *NFE2L2* expression much higher than that observed in Hs766T cell line (Figure 10B). Altogether, our data suggest that neither *CAT* nor *GPX2* expression would be a problem for the use of CLytA-DAAO in any of the three tumors analyzed. Regarding *NFE2L2*, CLytA-DAAO treatment would not be a problem either in pancreatic or colorectal carcinoma, however, in a percentage of glioblastoma patients, the expression level of these genes suggests a putative resistance to CLytA-DAAO treatment. The importance of ROS in carcinogenesis and treatment resistance in gliomas has been pointed out in the scientific literature [55]. One important relationship between the nuclear factor Nrf2 (*NFE2L2*) and mTOR has been pointed out to explain the complex role of ROS in gliomas. We are taking these data in consideration and increasing our glioblastoma patients’ samples and primary cultures in order to probe a cause–effect relationship between CLytA-DAAO activity and *NFE2L2* expression in glioblastoma.

In summary, it is necessary to expand the transcriptomic analysis by increasing the number of biopsies and analyzing the expression of more genes involved in sensitivity or resistance to the treatment with CLytA-DAAO. However, our data suggest that CLytA-DAAO immobilized in MNPs could be used as an anti-cancer therapy against pancreatic carcinoma, colorectal carcinoma, and glioblastoma. Moreover, we expect that this treatment would be effective in a high percentage of these patients.

## 4. Materials and Methods

### 4.1. Cell Culture

Hs766T, IMIM-PC-2 and RWP-1 pancreatic carcinoma cell lines and HT-29, SW-480 and SW-620 colorectal carcinoma cell lines were provided by the Instituto Municipal de Investigaciones Médicas (IMIM, Barcelona, Spain). HGUE-GB-18, HGUE-GB-37, HGUE-GB-39, and HGUE-GB-42 glioblastoma cell lines were established from primary cultures by our research group in Hospital General Universitario de Elche (HGUE, Elche, Spain) [56]. All cell lines used were maintained as previously described [11].

### 4.2. Patient Biopsies

Samples and data from patients included in this study were provided by the General University Hospital of Alicante (HGUA) Biobank, integrated in the Spanish National Biobanks Network and in the Valencian Biobanking Network, and were processed following standard operating procedures, with the appropriate approval of the Ethics and Scientific Committees.

### 4.3. Treatments

Except in dose-response analysis, the CLytA-DAAO standard concentration used in the different experiments was 2 U/mL with 1 mM D-Ala (Alfa Aesar, Thermo Fisher Scientific, Lancashire, UK) and 600 μM in H_2_O_2_ experiments (Merck Millipore, Burlington, MA, USA). The process to obtain CLytA-DAAO, its immobilization in magnetic nanoparticles (MNPs), and its subsequent quantification and activity measurement were previously described [11]. The MNPs used were DEAE-FluidMAG magnetic nanoparticles (Chemicell, Berlin, Germany), which have a diameter of 200 nm and are formed by a magnetite core and a DEAE-functionalized starch shell on its surface. Choline chloride (ACROS ORGANICS, Thermo Fisher Scientific, Geel, Belgium) was used for releasing the enzyme from MNPs.

CLytA-DAAO immobilization in the gold nanoparticles (GNPs) NITgold COOH-PEG 3000Da (30 nm) (NanoImmunotech, Zaragoza, Spain) was performed taking advantage of the gold affinity for sulfur. The pH of CLytA-DAAO was increased by dialysis in a sodium phosphate buffer (pH 8). Thus, deprotonating the sulfur from the thiol groups of cysteines. 500 μg of the dialyzed enzyme were incubated with 4.25 × 10^12^ of GNPs for 30 min with shaking. The remaining free enzyme was discarded by centrifugation.

For the enzyme trapping in alginate capsules, 100 μg of CLytA-DAAO were mixed with 1 mL of a sodium alginate solution. The mixture was then introduced into a syringe and slowly added to a 50 mM calcium chloride solution in stirring. The capsules formed were separated from the solution by filtration and washed with a 50 mM sodium phosphate buffer (pH 7).

### 4.4. Cell Death

The percentage of live and dead cells was determined using the Muse^®^ Count & Viability Kit (Luminex^®^, Austin, TX, USA) following the manufacturer’s instructions and results were measured in the Muse^®^ Cell Analyzer (Luminex^®^, Austin, TX, USA).

### 4.5. Proliferation Assays

To analyze de anti-proliferative effect generated by the different treatments methylthiazolyldiphenyl-tetrazolium bromide (MTT) assays were performed. Cells were seeded in 96-well plates (Sarstedt, Nümbrecht, Germany) and incubated at 37 °C and 5% CO_2_ for 24 h. Then, increasing concentrations of treatment were added in sextuplicate and the plate was incubated at the same conditions for 24 or 72 h according to the experiment. After the treatment time, 0.25 mg/mL MTT (Sigma-Aldrich, St. Louis, MO, USA) were added, and cells were maintained at 37 °C and 5% CO_2_ for 3 h. The medium was removed, 200 μL of dimethyl sulfoxide (DMSO) (Sigma-Aldrich, St. Louis, MO, USA) were added and the plates were shaken at room temperature for 30 min to dissolve the formazan crystals. Finally, the absorbance at 570 nm was measured on an Eon™ microplate spectrophotometer (BioTeK^®^, Winooski, VT, USA).

### 4.6. Cell Cycle Distribution

Cells were seeded in 6-well plates (Sarstedt, Nümbrecht, Germany) and incubated for 24 h at 37 °C and 5% CO_2_. Then, corresponding treatments were added, and cells were maintained in incubation for at least 24 h according to the experiment. Next, cells were trypsinized and fixed in cold ethanol (75%) for at least 1 h at −20 °C. Fixed cells were centrifuged, resuspended in 500 μL of a mixture formed by phosphate buffered saline (PBS) containing 0.5% Triton X-100, 25 μg/mL of RNase A (Serva, Heidelberg, Germany), and 25 μg/mL of propidium iodide (Sigma Aldrich, St. Louis, MO, USA) and incubated for 30 min at room temperature protected from light. Finally, cell cycle distribution according to the DNA content, was determined using a BD FACSCantoTM flow cytometer (Becton Dickinson & Co., Franklin Lakes, NJ, USA).

### 4.7. Intracellular Free Radicals’ Measurement

Cells were seeded in opaque 96-well plates (Sigma-Aldrich, St. Louis, MO, USA) and incubated at 37 °C and 5% CO_2_ for 24 h. Then, cells were treated with 2 U/mL CLytA-DAAO and 1 mM D-Ala or 600 μM H_2_O_2_ for 20–120 min. Along with the treatment, 10 μg/mL 2′,7′-dichlorodihydrofluorescein diacetate (H2DCF-DA) (Sigma-Aldrich, St. Louis, MO, USA) were added to cells. H2DCF-DA is a probe able to emit fluorescence when is bound to free radicals inside cells. For each treatment time used, a control containing only the probe was added. After the different treatment times, medium was removed, and plates were washed with PBS. Finally, the plate reader POLARstar Omega (BMG Labtech, Ortenberg, Germany) was used to measure fluorescence using an excitation wavelength of 485 nm and an emission wavelength of 520 nm.

### 4.8. Gene Expression Analysis

Most of the biopsies were obtained from frozen tissue in Optimal Cutting Temperature (OCT) medium since the quality and quantity of the extracted nucleic acids is higher than the obtained from fixed tissue. However, due to lack of material, the study was completed with some paraffin tissue samples.

To isolate RNA from frozen tissue, initially OCT medium was removed, and the tissue was chopped and homogenized in a lysis mix of RLT Plus Buffer with β-ME (1:100). In large samples, to achieve complete degradation of tissue, sonication was performed and RNeasy^®^ Plus Mini Kit (Qiagen, Hilden, Germany) was used. To extract RNA from the paraffin-fixed samples, initially paraffin was dissolved in 600 μL mineral oil and samples were incubated for 2 min at 95 °C and centrifuged to remove the oil paraffin mixture, then, the RNeasy^®^ FFPE kit (Qiagen, Hilden, Germany) was used. In both types of sample, the RNA obtained was retrotranscribed into cDNA using the High-Capacity cDNA Reverse Transcription Kit (Applied Biosystems, Foster City, CA, USA). RNeasy^®^ Plus Mini Kit, RNeasy^®^ FFPE kit and High-Capacity cDNA Reverse Transcription Kit were used following the manufacturer’s instructions.

Finally, 4 μL of cDNA samples were mixed with 10 μL NZYSpeedy qPCR Probe Master Mix (2×), ROX plus (NZYtech, Lisbon, Portugal) and 1 μL predesigned TaqMan^®^ Gene Expression Assays (Applied Biosystems, Foster City, CA, USA) and 7300 Real Time PCR System was used to perform quantitative polymerase chain reaction (qPCR). The Taqman Gene Expression Assays used to measure the expression of the following genes were CAT (Hs00156308_m1), NFE2L2 (Hs00975961_g1), GPX2 (Hs01591589_m1) and 18S rRNA (Hs03003631_g1) as endogenous control.

### 4.9. Statistical Analysis

All data are represented in graphs as the mean ± standard deviation (SD) of at least three independent data. Statistical analysis was performed using the GraphPad Prism version 5 (GraphPad Software Inc., San Diego, CA, USA), as described in a previous article [11].

## 5. Conclusions

- Our results demonstrate that CLytA-DAAO bound to MNPs is more effective at inducing cytotoxicity than free CLytA-DAAO in pancreatic carcinoma, colorectal carcinoma, and glioblastoma cell models.
- The higher effect is due to the fact that immobilization increases the stability of the enzyme at 37 °C, maintaining its catalytic activity for a longer time.
- The enzyme is bound to the MNPs by a non-covalent immobilization system, which allows the release of the enzyme once it has reached its target by the external addition of choline.
- The cytotoxic effect induced by CLytA-DAAO is due to the prolonged production of ROS over time and is not comparable to adding H_2_O_2_ directly.
- Expression analysis of genes, which we have previously found to be related to CLytA-DAAO resistance, performed in biopsies as well as data extracted from UALCAN, suggest that CLytA-DAAO bound to MNPs could be effective as an anti-cancer therapy in a wide range of patients from pancreatic carcinoma, colorectal carcinoma, and glioblastoma. However, *NFE2L2* expression has to be taken in consideration, especially in glioblastoma patients.

## Figures and Tables

**Table 1:** Anti-proliferative percentage of 0.025 U/mL and 0.25 U/mL CLytA-DAAO, free or bound to MNPs, with 1 mM D-Ala and IC50 value on pancreatic carcinoma (PC), colorectal carcinoma (CRC), and glioblastoma (GBM) cell lines. Cells were treated with a concentration range between 0.025–2 U/mL CLytA-DAAO and 1 mM D-Ala for 72 h.

Origin	Cell Line	%Inhibition (0.025 U/mL)		%Inhibition (0.25 U/mL)		IC50 (U/mL)	
		Free	MNPs	Free	MNPs	Free	MNPs
PC	IMIM-PC-2	8.6 ± 10.5	17.0 ± 10.1	60.5 ± 8.5	73.4 ± 3.5	0.19 ± 0.04	0.13 ± 0.02
	RWP-1	7.5 ± 6.9	5.9 ± 9.5	61.8 ± 11.1	80.2 ± 4.3	0.19 ± 0.02	0.14 ± 0.03
	Hs766T	33.8 ± 14.4	47.7 ± 3.1	62.7 ± 13.5	62.5 ± 14.6	0.10 ± 0.02	0.05 ± 0.02
CRC	SW-480	16.1 ± 8.1	26.9 ± 6.9	45.6 ± 11.3	67.7 ± 7.0	0.17 ± 0.03	0.09 ± 0.01
	SW-620	23.6 ± 5.4	41.7 ± 5.8	57.3 ± 3.3	78.1 ± 6.4	0.16 ± 0.02	0.05 ± 0.01
	HT-29	0.0 ± 5.6	33.9 ± 8.5	22.4 ± 8.1	64.9 ± 8.1	0.68 ± 0.10	0.10 ± 0.02
GBM	HGUE-GB-18	15.4 ± 8.9	34.3 ± 7.9	24.4 ± 8.4	81.4 ± 8.7	0.50 ± 0.08	0.07 ± 0.01
	HGUE-GB-37	22.2 ± 5.8	37.8 ± 4.9	30.7 ± 7.6	75.7 ± 3.9	0.38 ± 0.06	0.07 ± 0.01
	HGUE-GB-39	35.8 ± 7.9	50.1 ± 1.5	41.0 ± 8.4	81.2 ± 4.5	0.27 ± 0.06	0.03 ± 0.01
	HGUE-GB-42	7.8 ± 6.4	30.8 ± 6.1	26.9 ± 9.0	72.9 ± 7.1	0.54 ± 0.05	0.08 ± 0.01

**Table 2:** IC50 value of CLytA-DAAO, free and bound to MNPs, on pancreatic and colorectal carcinoma cell lines. Cells were treated with a concentration range between 0.025-2 U/mL CLytA-DAAO and 1 mM D-Ala for 24 h.

Cell Line	IC50 (U/mL)	
	Free	MNPs
IMIM-PC-2	1.26 ± 0.17	0.11 ± 0.03
RWP-1	0.70 ± 0.08	0.12 ± 0.02
SW-480	1.12 ± 0.21	0.24 ± 0.08
SW-620	0.78 ± 0.30	0.17 ± 0.08

**Table 3:** IC50 value of H_2_O_2_ on pancreatic carcinoma (PC), colorectal carcinoma (CRC) and glioblastoma (GBM) cell lines. Cells were treated with a concentration range between 50 and 600 μM H_2_O_2_ for 72 h.

Origin	Cell Line	IC50 (μM)
PC	Hs766T	358.0 ± 76.5
	IMIM-PC-2	222.1 ± 55.9
	RWP-1	83.71 ± 1.92
CRC	SW-480	54.1 ± 2.77
	SW-620	145.3 ± 17.0
GBM	HGUE-GB-37	776.6 ± 157.5
	HGUE-GB-39	362.3 ± 95.5

## Supplementary Materials

The following are available online at https://www.mdpi.com/1422-0067/22/3/1477/s1. Figure S1: Cells accumulation in SubG1 and G2/M phases after a CLytA-DAAO, either free or bound to MNPs, and D-Ala treatment in RWP-1 pancreatic carcinoma cell line and SW-620 colorectal carcinoma cell line, Figure S2: Differential effects between H_2_O_2_ and CLytA-DAAO treatment in IMIM-PC-2 pancreatic carcinoma cell line, Figure S3: Gene expression analysis in patient samples performed with the UALCAN platform.
