# Supplementary material for: CLytA-DAAO Chimeric Enzyme Bound to Magnetic Nanoparticles. A New Therapeutical Approach for Cancer Patients?
Source: Int J Mol Sci. 2021 Feb 2;22(3):1477. doi: 10.3390/ijms22031477 (PMC7867295; doi:10.3390/ijms22031477)

Figure S1. Cells accumulation in SubG1 and G2/M phases after a CLytA-DAAO, either free or bound to MNPs, and D-Ala treatment in RWP-1 pancreatic carcinoma cell line and SW-620 colorectal carcinoma cell line. Cells were treated with 2 U/mL CLytA-DAAO and 1 mM D-Ala for a short time (15-60 min) and then, treatment was removed replacing the medium. Cells were incubated for 24 h from the treatment addition were completed. Graph shows the percentage of cells ± SD in subG1 and G2/M phases (n ≥ 3). * indicates a p-value < 0.05 and ** < 0.01.

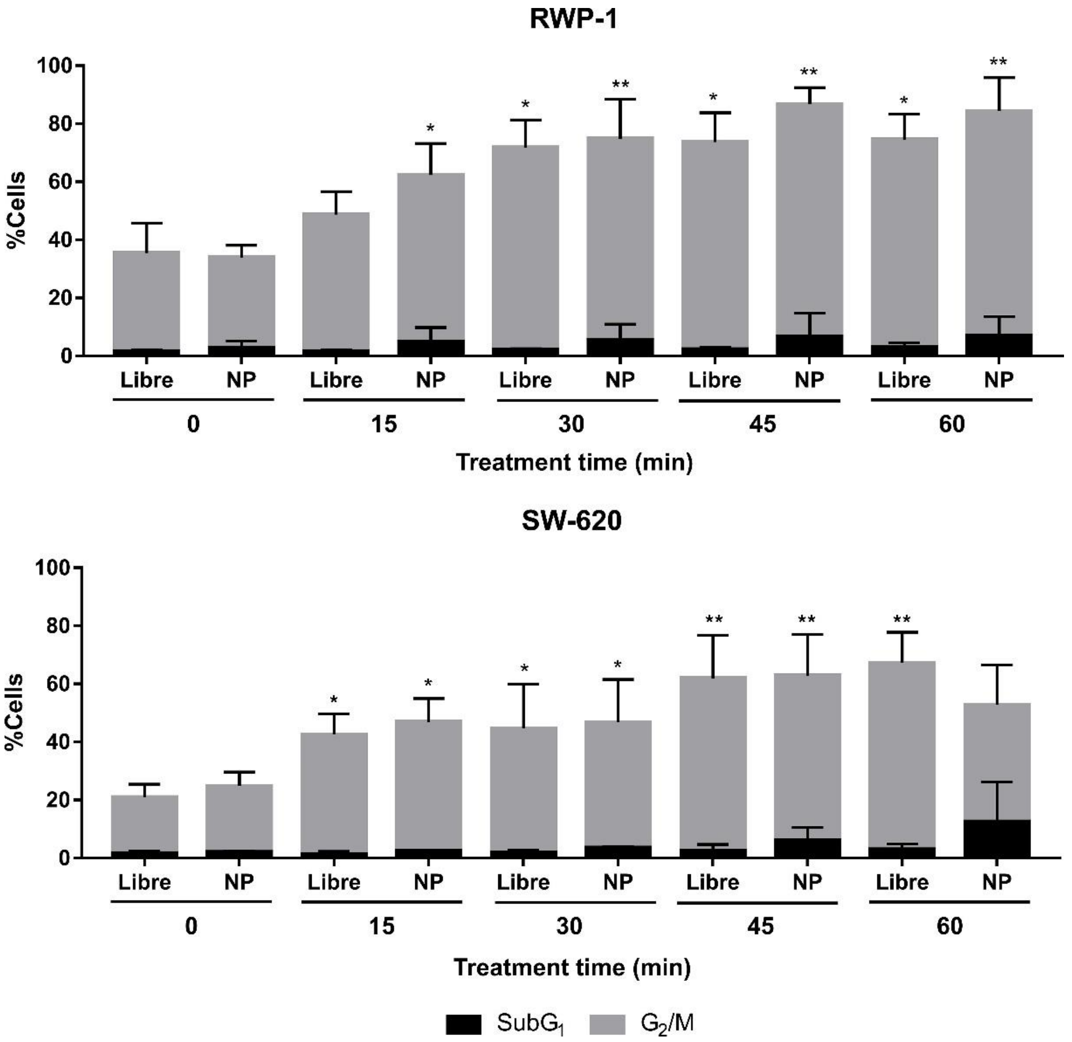

Figure S2. Differential effects between H_2O_2 and CLytA-DAAO treatment in IMIM-PC-2 pancreatic carcinoma cell line. A. Intracellular ROS increase after a treatment with 2 U/mL CLytA-DAAO and 1 mM D-Ala or 600 µM H_2O_2 for 20-120 min. Free radical production was determined through $DCFH_2$-DA probe and each treatment time had a control untreated that only contained the probe. Graph shows the fold change (FC) ± SD of fluorescent intensity with respect to the control ($n \geq 6$). **B.** Variations in cell cycle distribution after a treatment with 2 U/mL CLytA-DAAO and 1 mM D-Ala or 600 µM H_2O_2 for 24 h. Graph shows the cells percentage ± SD in each phase of cell cycle after subtracting the cells percentage in the control untreated ($n \geq 3$). **C.** Plasmatic membrane rupture after a treatment with 2 U/mL CLytA-DAAO and 1 mM D-Ala or 600 µM H_2O_2 for 24 h. Graph represents cell death percentage (mean ± SD) after subtracting cell death in the control untreated ($n \geq 3$). ** indicates a p-value < 0.01 and *** a p-value < 0.001.

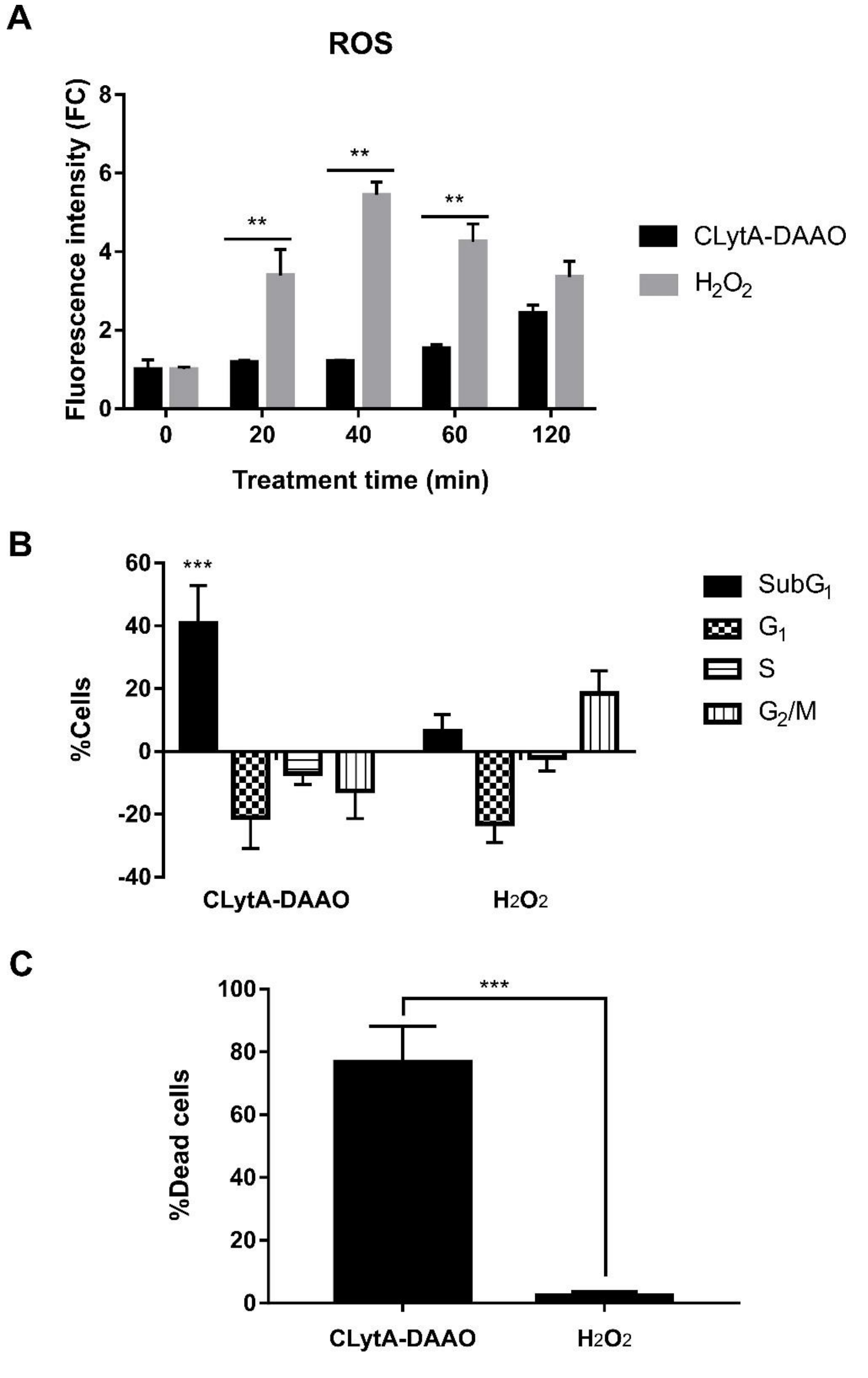

Figure S3. Gene expression analysis in patient samples performed with the UALCAN platform. Graphs show the expression of CAT (**A**), NFE2L2 (**B**) and GPX2 (**C**) in samples TGCA samples from normal tissue and tumoral tissue of colon adenocarcinoma (COAD), glioblastoma (GBM) and pancreatic adenocarcinoma (PAAD). * indicates a p-value < 0.05 and *** a p-value < 0.001.

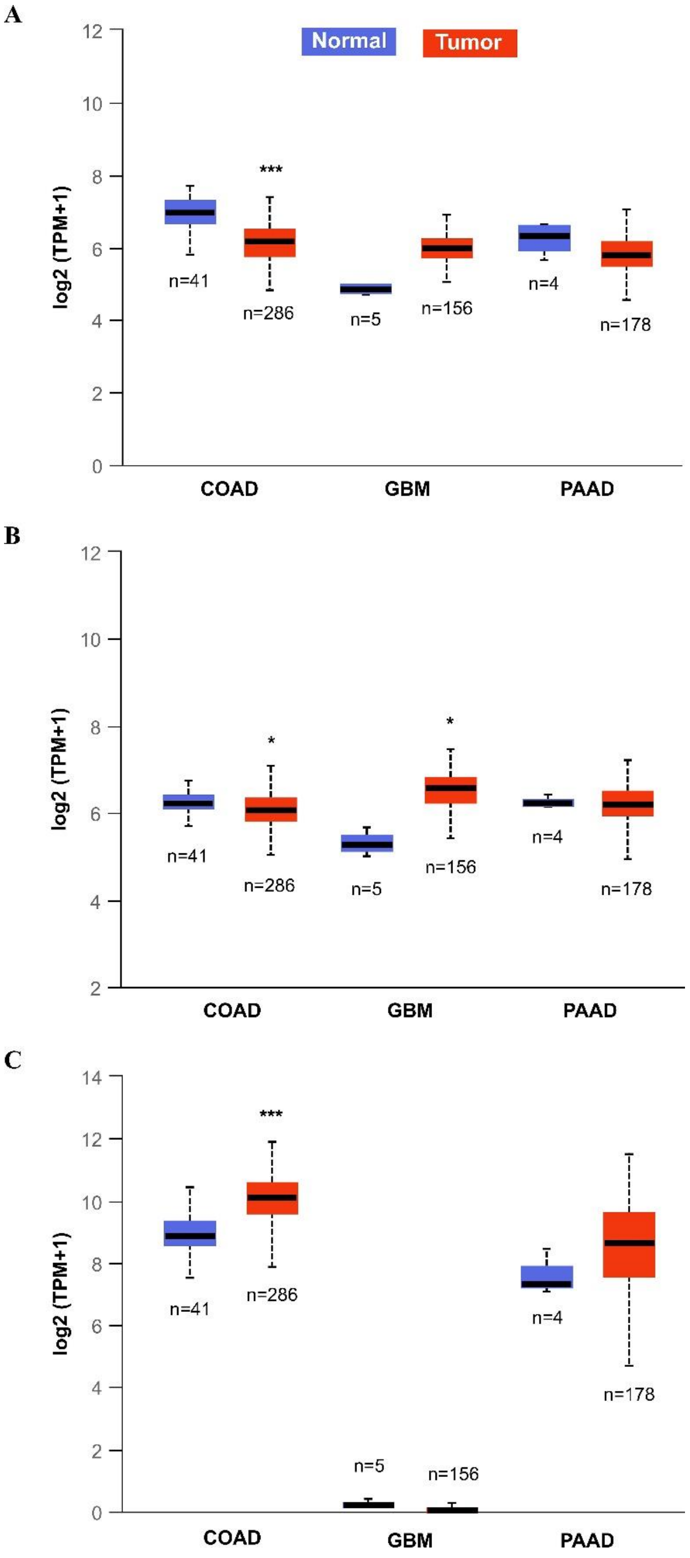

Supplement: Supplementary file 1 [file ijms-22-01477-s001.pdf]
